# Supplementary material for: Galectin-9 and Interferon-Gamma Are Released by Natural Killer Cells upon Activation with Interferon-Alpha and Orchestrate the Suppression of Hepatitis C Virus Infection
Source: Viruses. 2022 Jul 14;14(7):1538. doi: 10.3390/v14071538 (PMC9317111; doi:10.3390/v14071538)
Supplement: Supplementary file 1 [file viruses-14-01538-s001.zip › viruses-1680226-supplementary.pdf]

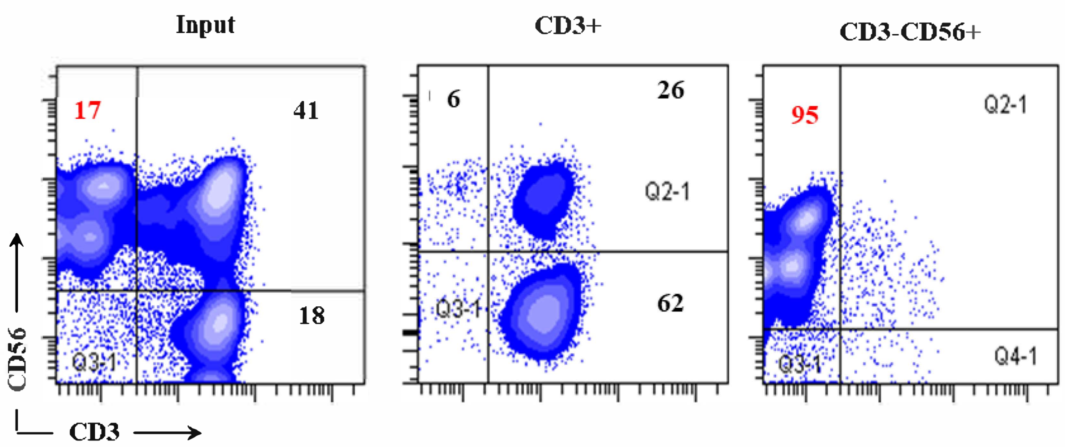

Supplementary Figure S1. Flow cytometric scatter dot-plot images of CD3 and CD56 expression in unsorted (*left panel*) or sorted CD3^{+} (*middle panel*) and CD3^{-}CD56^{+} (*right panel*) cell populations obtained from the buffy coats of liver perfusates. Numbers represent percentages of positive cells.

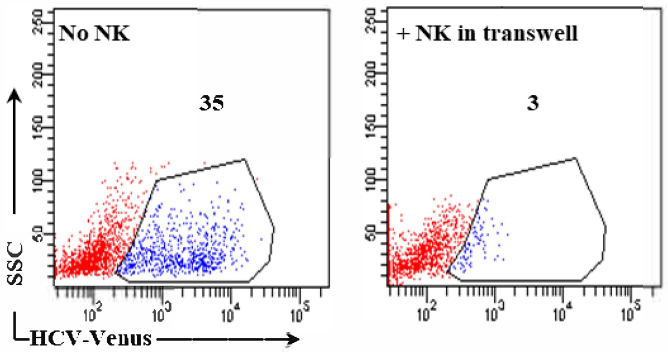

Supplementary Figure S2. Representative flow cytometry scatter dot-plot images of Venus expression (blue dots) in HCV-Venus Huh7.5 target cells untreated (left image) or co-cultured in transwells with IFNα-NKs (right image). Numbers represents the percentages of positive cells. SSC, side scatter.

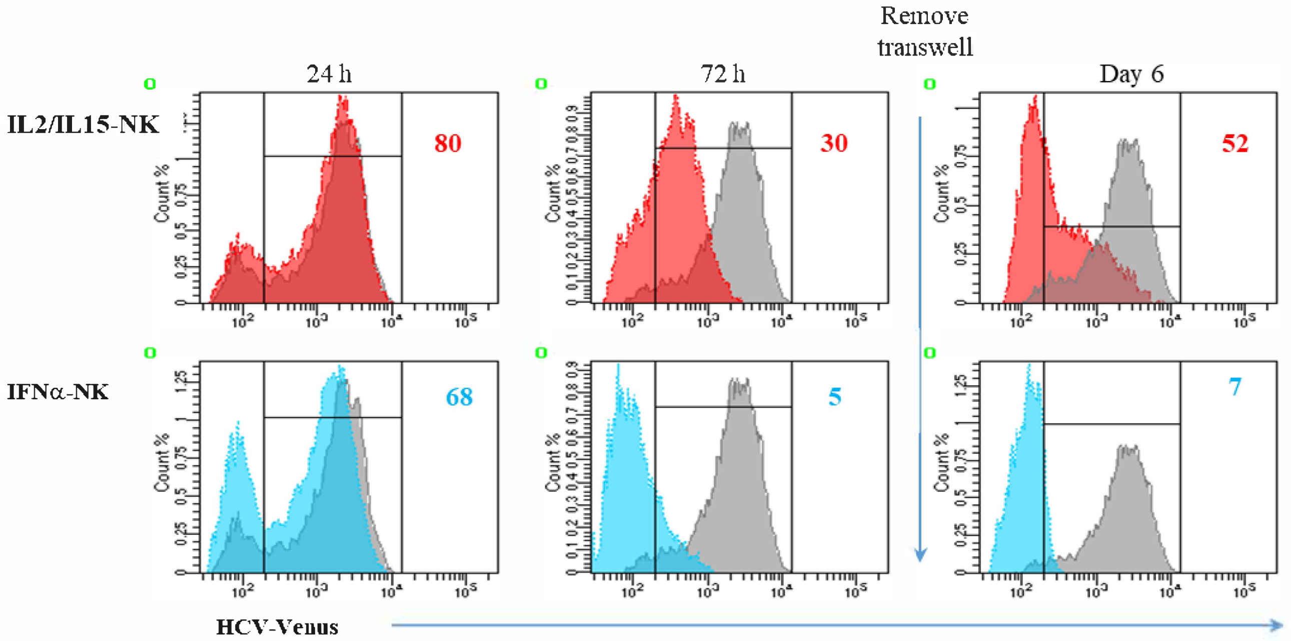

Supplementary Figure S3. Representative flow cytometry histograms of Venus expression (HCV infection) in Huh7.5 target cells which were co-cultured in transwells with IL2/IL15-NKs (*top images*) or IFNα-NKs (*bottom images*). Transwells were removed 72 hours after the start of co-culture and target cells were maintained for three additional days (until Day 6) in fresh media. Plots show Venus expression in untreated target cells (gray histograms) versus treated cells (red or blue histograms). Numbers represents the percentages of Venus^{+} cells.

Supplementary Figure S4

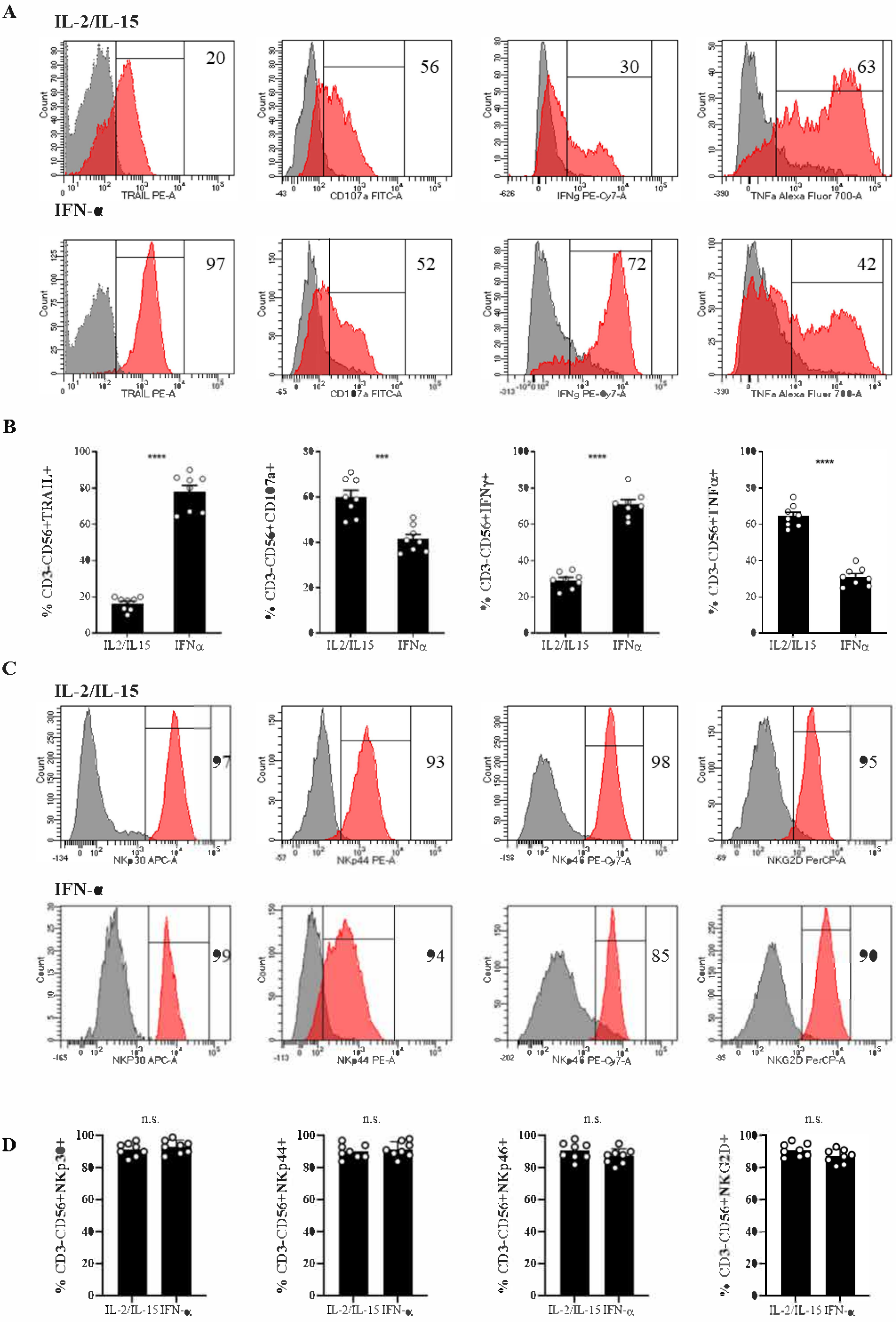

Supplementary Figure S4. (A) Representative flow cytometry histograms of gated $CD3^{-}CD56^{+}$ NK cells showing expression of TRAIL, CD107a or intracellular IFN-γ and TNF-α following overnight stimulation with IL-2/IL-15 or IFN-α. Plots show specific antibody staining of unstimulated (open histogram) versus stimulated NK cells (black histogram). Numbers represent percentages of positive cells. Histograms are representative of one biological sample out of eight stained in the same conditions. **(B)** Bar graphs represent expression (percentages) of TRAIL, CD107a, IFN-γ or TNF-α in $CD3^{-}CD56^{+}$ NK cells stimulated as in **A**. Each dot represents the average of three technical replicates from each donor (n=8). Mean values ± SD are shown. ***$P < 0.001$, ****$P < 0.0001$. **(C)** Representative flow cytometry histograms of gated $CD3^{-}CD56^{+}$ NK cells showing expression of NKp30, NKp44, NKp46 and NKG2D following overnight stimulation with IL-2/IL-15 or IFN-α. Plots show specific antibody staining of unstimulated (gray histogram) versus stimulated NK cells (red histogram). Numbers represent percentages of positive cells. Histograms are representative of one biological sample out of eight stained in the same conditions. **(D)** Bar graphs represent expression (percentages) of NKp30, NKp44, NKp46 and NKG2D in $CD3^{-}CD56^{+}$ NK cells stimulated as in **C**. Each dot represents the average of three technical replicates from each donor (n=8). Mean values ± SD are shown. n.s. non significant.

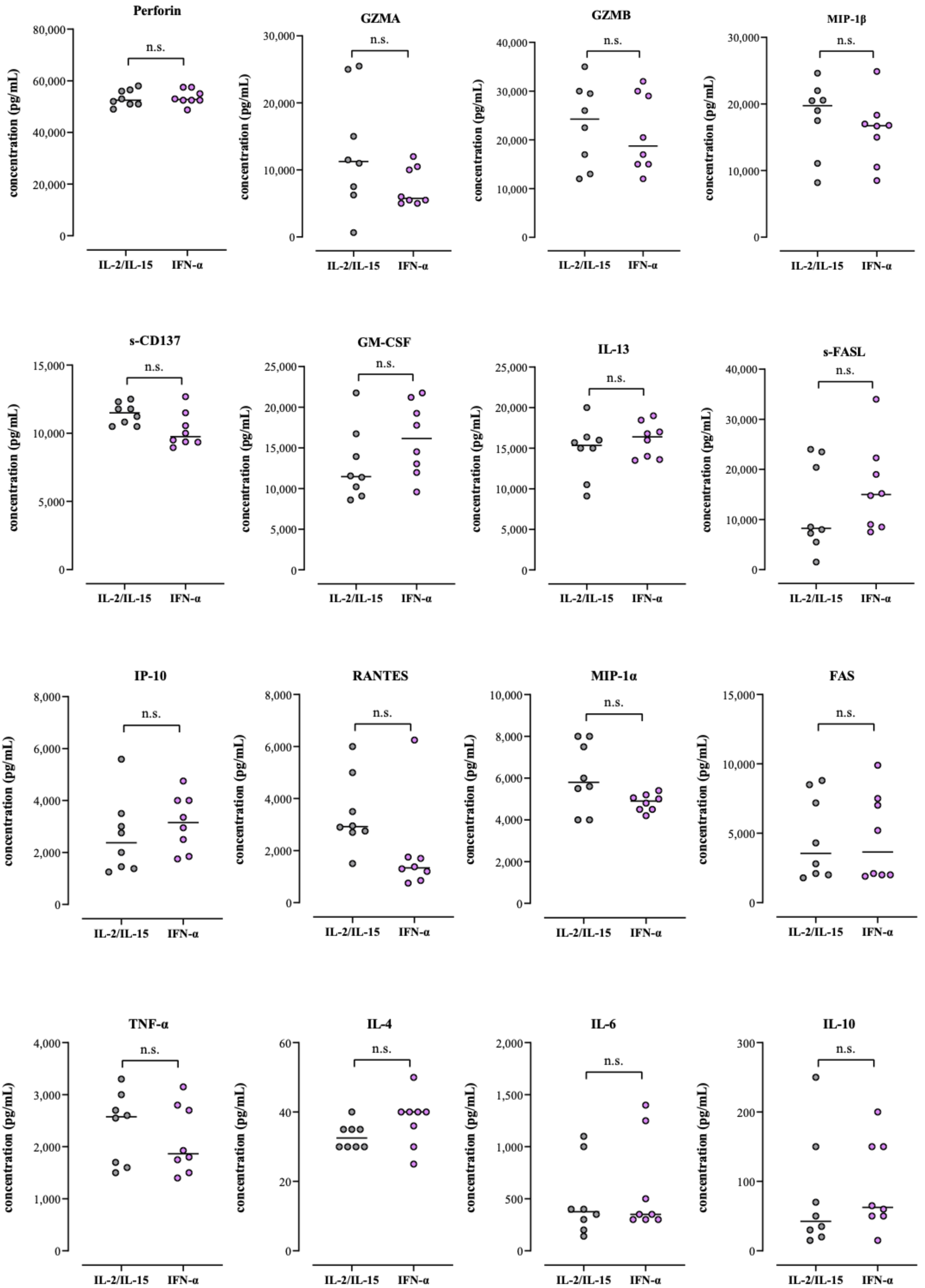

Supplementary Figure S5. Dot plots of cytokine expression (pg/mL) in the conditioned media of NK cells activated overnight with IL-2/IL-15 (gray) or IFN-α (lilac). Each represents a donor (n=8). Means are shown. Statistical analysis was done using Student's t-test. n.s., non-significant.

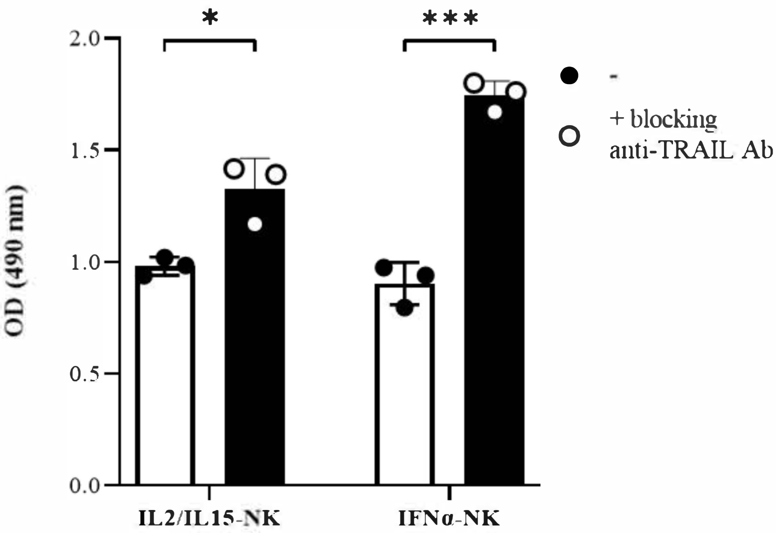

Supplementary Figure S6. MTS cell viability assay of HCV-Huh7.5 cells co-cultured in transwell with IL2/IL15-NK or IFNα-NKs in the presence of neutralizing anti-TRAIL antibody. Statistical analysis was done using Student's *t*-test. $^{*}P < 0.05$, $^{***}P < 0.001$.

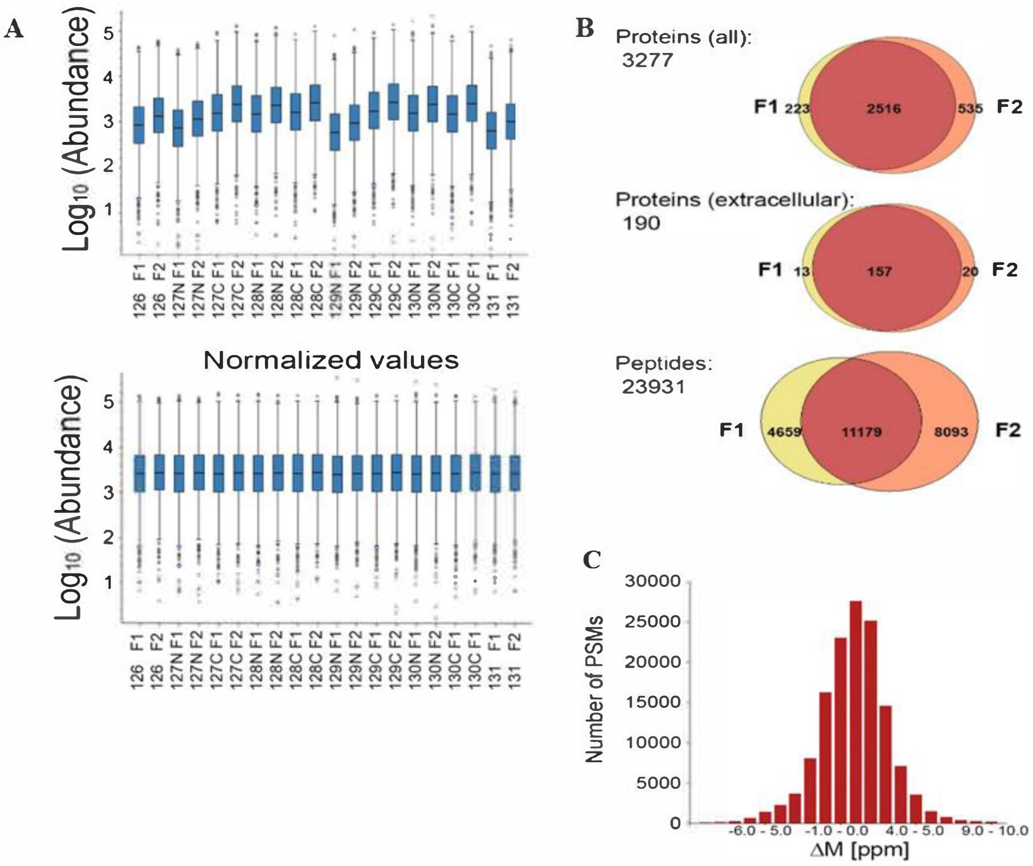

Supplementary Figure S7. (A) Box plots of protein abundances before and after normalization, showing similar ranges of total protein amount in replicates and efficiency of normalization. **(B)** Venn diagrams of total proteins identified and quantified using F1 and F2 fractions (*top*); total proteins filtered by Gene Ontology (GO) "extracellular" term (*middle*); total number of peptides identified and quantified using F 1 and F2 fractions (*bottom*). **(C)** Distribution of Delta mass (ΔM) of peptide spectrum matches (PSM) expressed in parts per million (ppm).

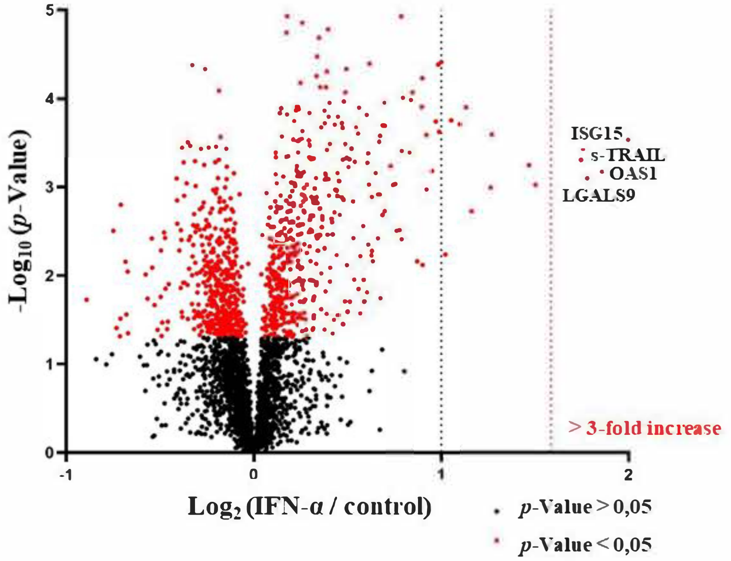

Supplementary Figure S8. Volcano plot of proteins (n=3,277) in IFNα-NK secretome as compared to untreated NK cells as identified and quantified by MS-based quantitative proteomics. Plotted along the x axis is the mean of log2 fold-change; along the y axis is the negative logarithm to the base 10 of P values from Student's t-test. The vertical black dashed line reflects a fold change ≥ 2.0 while the vertical red dashed line reflects fold change ≥ 3.0. Significant hits ($P < 0.05$) are depicted in red. The annotated dots are the four data points that have the largest distance from the origin and are above the fold change ≥ 3.0 and P values ≤ 0.05 thresholds.

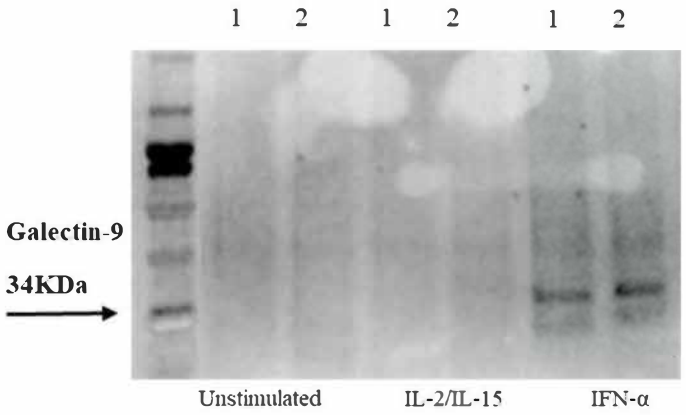

Supplementary Figure S9. Representative western blot image of galectin-9 (34kDa) in the secretome of NK cells treated overnight with IL-2/IL-15 or IFN-α. Data from two individuals (indicated as 1 and 2) are shown. Unstimulated cells were used as negative control.

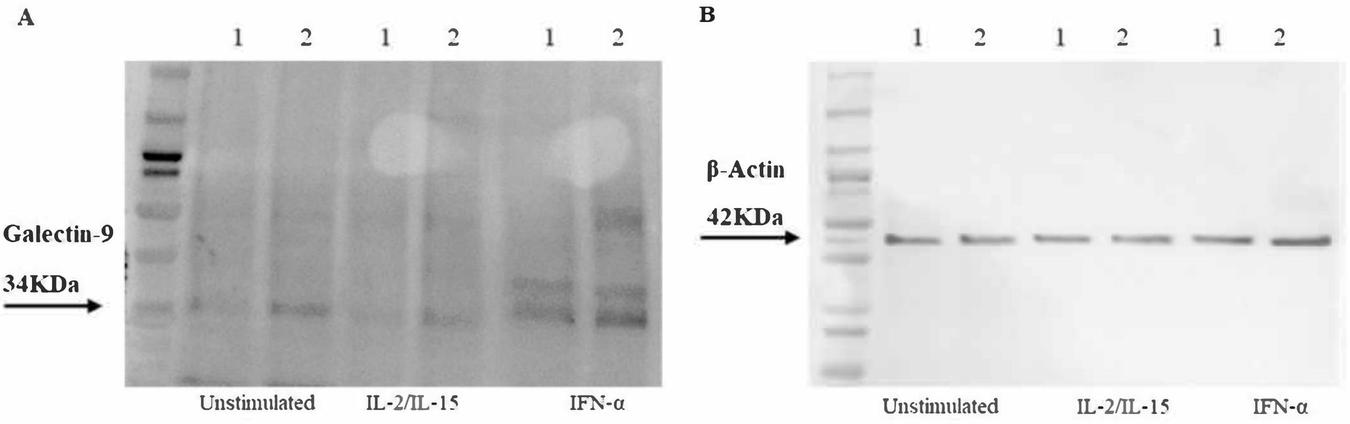

Supplementary Figure S10. Representative western blot images of galectin-9 (34kDa) **(A)** or β-actin (42kD) **(B)** in the lysate of NK cells treated overnight with IL-2/IL-15 or IFN-α. Data from two individuals (indicated as 1 and 2) are shown. Unstimulated cells were used as negative control.

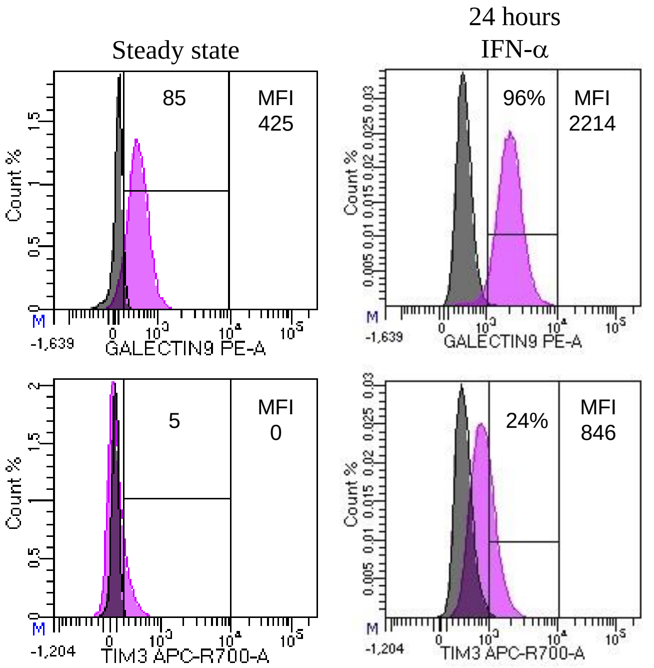

Supplementary Figure S11. Representative flow cytometry histograms of intracellular galectin-9 (*top panels*) or TIM-3 (*bottom panels*) expression in IFNα-NKs at 24 hours. Plots show isotype control IgG (gray histogram) versus specific antibody staining (purple histogram). Mean fluorescence intensity (MFI) values and percentages of positive cells are shown.

Supplementary Figure S12. RMSD plot of MD simulations performed on the complexes involving PDB structures 6MEK and 2ZHM (on the left), and the structures from PDB 6MEJ and 2ZHN (on the right).

2ZHM-6MEK Complex	2ZHN-6MEJ Complex

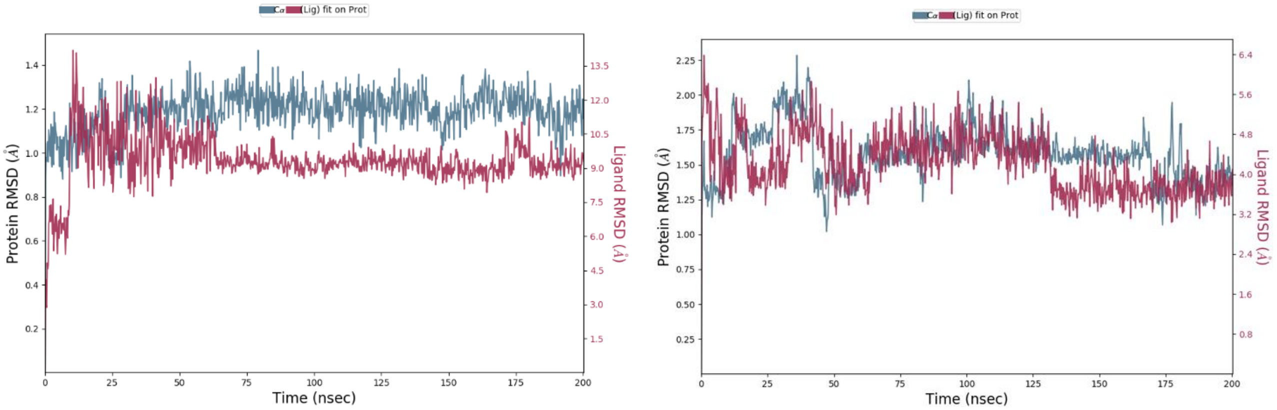

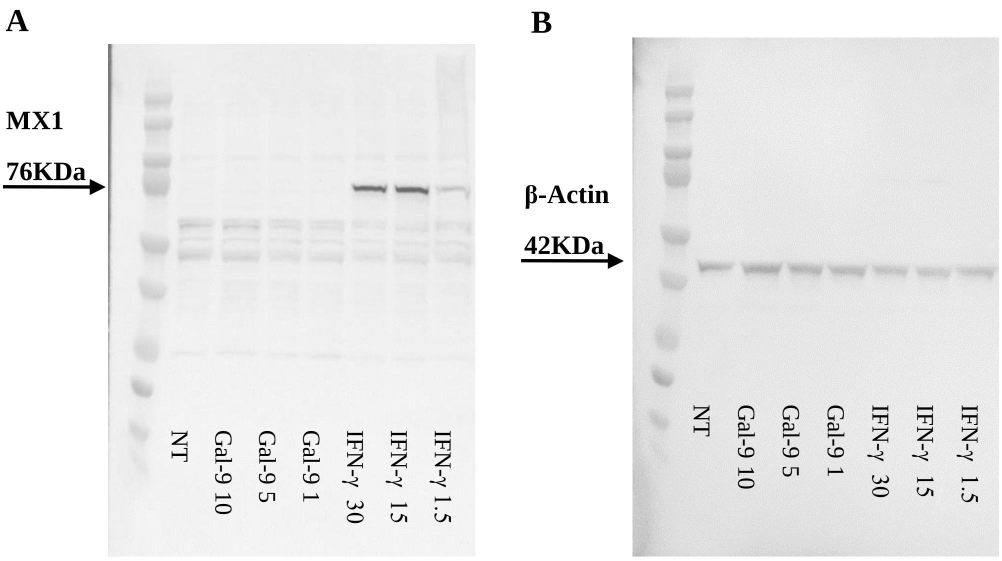

Supplementary Figure S13. Representative western blot images of MX1 (75kDa, **A)** or β-actin (42kD, **B**) in lysates of HCV-Huh7.5 cells following stimulation with decreasing doses of galectin-9 (1-10 ng/mL) or IFN-γ (1.5-30 ng/mL).

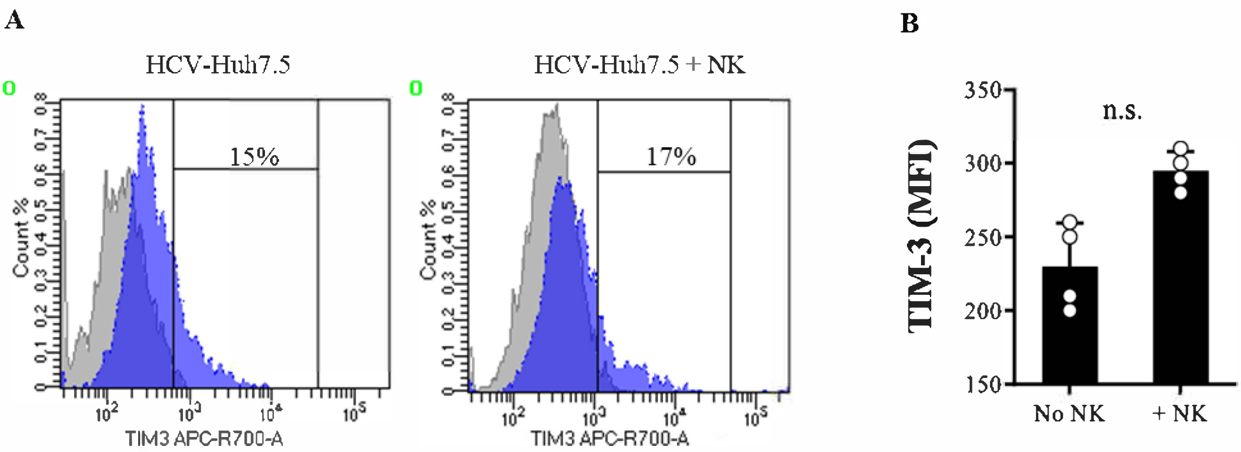

Supplementary Figure S14. (A) Representative flow cytometry histograms of TIM-3 expression in HCV-Huh7.5 cells co-cultured with IFNα-NKs. Percentages of positive cells are shown **(B)**. Bar graphs represent mean fluorescence intensity (MFI) values of TIM-3 positive signal in cells as in **A**. Each dot represents the average of three technical replicates from one donor (*n*=4). Mean values ± SD are shown. (n.s., non-significant).

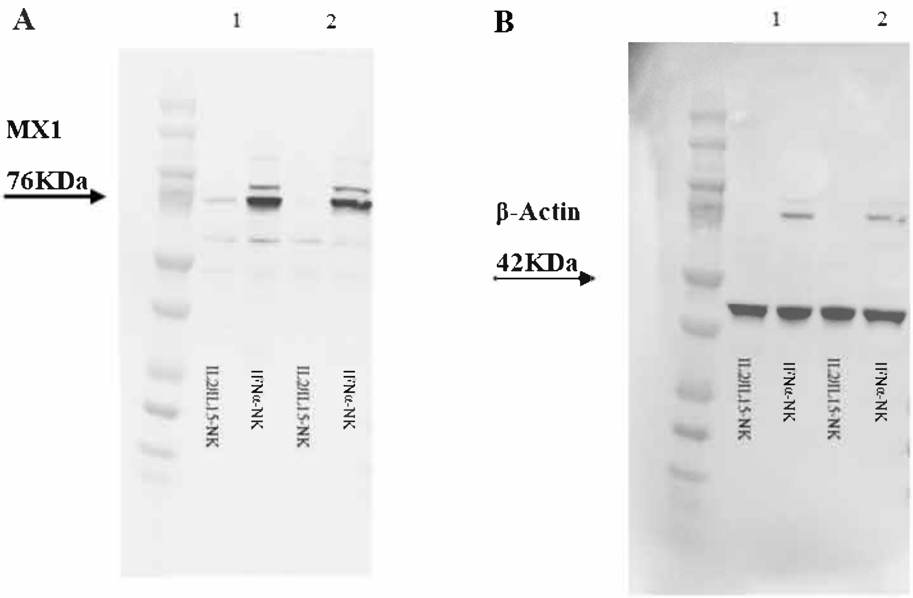

Supplementary Figure S15. Representative western blot images of MX1 (75kDa, **A**) or β-actin (42kD, **B**) in lysates of HCV-Huh7.5 cells following stimulation with IL2/IL15-NK or IFNα-NK

Table S1. Interactions established between Gal-9 CRD amino acids and HCV E2 residues within the docking poses of the complexes 2ZHM-6MEK and 2ZHN-6MEJ.

2ZHM-6MEK COMPLEX			2ZHN-6MEJ COMPLEX		
Gal 9 CRD	HCV E2	Specific Interactions	Gal 9 CRD	HCV E2	Specific Interactions
Gln 121	Asn 434	1 H-bond	Arg 125	Ser 432	1 H-bond
Gln 94	Glu 454	1 H-bond	Phe 123	Ser 432	1 H-bond
Lys 88	Thr 435	1 H-bond	Arg 87	Arg 483	1 H-bond
Arg 87	Thr 558	1 H-bond	Arg 87	Asp 481	1 H-bond
Arg 87	Thr 435	1 H-bond	Glu 86	Ser 432	2 H-bonds
Glu 86	Ser 432	1 H-bond	Glu 86	Trp 437	1 H-bond
Tyr 71	Phe 560	1 π-stacking	Glu 85	Thr 558	1 H-bond
			Ash 68	Arg 455	1 H-bond
			Arg 44	Asp 481	1 H-bond + 1 salt bridge

Table S2. Interactions between Gal-9 CRD and HCV E2 amino acids retrieved for the MD cluster centroids of the complexes 2ZHM-6MEK and 2ZHN-6MEJ.

2ZHM-6MEK		CLUSTER 1	CLUSTER 2	CLUSTER 3	CLUSTER 4	CLUSTER 5	CLUSTER 6	CLUSTER 7	CLUSTER 8	CLUSTER 9	CLUSTER 10	
Gal9 CRD	HCV E2	Interaction	Interaction	Interaction	Interaction	Interaction	Interaction	Interaction	Interaction	Interaction	Interaction	Frequency
Lys 95	Glu 454	1 H-bond	1 H-bond	1 H-bond	1 H-bond	1 H-bond	1 H-bond	-	1 H-bond	1 H-bond	1 H-bond	9
Lys 95	Pro 453	-	1 H-bond	-	1 H-bond	-	1 H-bond	-	-	-	-	3
Gln 94	Glu 454	-	-	-	-	-	-	1 H-bond	-	-	-	1
His 90	Asp 448	1 H-bond	1 H-bond	1 H-bond	1 H-bond	-	1 H-bond	1 H-bond	1 H-bond	1 H-bond	1 H-bond	9
Thr 89	Arg 455	-	-	-	-	-	-	-	-	1 H-bond	1 H-bond	2
Lys 88	Leu 433	1 H-bond	1 H-bond	1 H-bond	1 H-bond	1 H-bond	1 H-bond	1 H-bond	1 H-bond	1 H-bond	1 H-bond	10
Lys 88	Asn 434	-	-	-	1 H-bond	-	-	-	1 H-bond	-	-	2
Arg 87	Thr 558	1 H-bond	1 H-bond	1 H-bond	1 H-bond	1 H-bond	1 H-bond	1 H-bond	1 H-bond	1 H-bond	1 H-bond	10
Glu 86	Ser 432	1 H-bond	-	1 H-bond	1 H-bond	1 H-bond	1 H-bond	1 H-bond	1 H-bond	1 H-bond	1 H-bond	9
Tyr 71	Phe 560	-	-	-	1 π-stacking	-	-	-	1 π-stacking	-	-	2
Gly 70	Arg 455	1 H-bond	-	1 H-bond	1 H-bond	1 H-bond	1 H-bond	-	1 H-bond	1 H-bond	-	7
Gly 69	Arg 455	1 H-bond	1 H-bond	1 H-bond	1 H-bond	1 H-bond	1 H-bond	2 H-bonds	1 H-bond	1 H-bond	1 H-bond	10
2ZHN-6MEJ		CLUSTER 1	CLUSTER 2	CLUSTER 3	CLUSTER 4	CLUSTER 5	CLUSTER 6	CLUSTER 7	CLUSTER 8	CLUSTER 9	CLUSTER 10	
Gal9 CRD	HCV E2	Interaction	Interaction	Interaction	Interaction	Interaction	Interaction	Interaction	Interaction	Interaction	Interaction	Frequency
Arg 125	Ser 432	-	-	-	-	1 H-bond	-	1 H-bond	-	-	1 H-bond	3
Phe 123	Ser 432	1 H-bond	2 H-bonds	2 H-bonds	1 H-bond	1 H-bond	2 H-bonds	1 H-bond	1 H-bond	1 H-bond	1 H-bond	10
Gln 121	Asn 434	1 H-bond	-	-	1 H-bond	-	-	-	-	1 H-bond	1 H-bond	4
Thr 89	Asn 448	1 H-bond	1 H-bond	-	-	-	-	-	-	-	-	2
Arg 87	Thr 558	-	-	-	-	-	-	-	-	1 H-bond	-	1
Glu 86	Leu 433	1 H-bond	1 H-bond	1 H-bond	1 H-bond	1 H-bond	1 H-bond	1 H-bond	1 H-bond	1 H-bond	1 H-bond	10
Glu 86	Ser 432	1 H-bond	1 H-bond	-	2 H-bonds	1 H-bond		2 H-bonds	1 H-bond	2 H-bonds	1 H-bond	8
Glu 85	Thr 558	1 H-bond	-	-	1 H-bond	1 H-bond	1 H-bond	1 H-bond	1 H-bond	1 H-bond	1 H-bond	8
Asn 68	Arg 455	-	-	-	1 H-bond	-	-	-	-	-	-	1

Table S3. Most frequent and stable hydrogen-bonds established between Gal9 CRD and HCV E2 residues during the two MD simulations performed on 2ZHM-6MEK and 2ZHN-6MEJ complexes.

MD on 2ZHM-6MEK complex		MD on 2ZHN-6MEJ complex	
Gal9 CRD	HCV E2	Gal9 CRD	HCV E2
Lys 95	Glu 454	Phe 123	Ser 432
His 90	Asp 448	Glu 86	Leu 433
Lys 88	Leu 433	Glu 86	Ser 432
Arg 87	Thr 558	Glu 85	Thr 558
Glu 86	Ser 432		
Gly 70	Arg 455		
Gly 69	Arg 455		
